# Molecular Cloning and Sequence Analysis of the cDNAs Encoding Toxin-Like Peptides from the Venom Glands of Tarantula *Grammostola rosea*

**DOI:** 10.1155/2012/731293

**Published:** 2012-02-29

**Authors:** Tadashi Kimura, Seigo Ono, Tai Kubo

**Affiliations:** ^1^Molecular Neurophysiology Group, Neuroscience Research Institute, National Institute of Advanced Industrial Science and Technology (AIST), Central 6, 1-1-1 Higashi, Tsukuba, Ibaraki 305-8566, Japan; ^2^United Graduate School of Drug Discovery and Medical Information Science, Gifu University, 1-1 Yanagido, Gifu 501-1193, Japan

## Abstract

Tarantula venom glands produce a large variety of bioactive peptides. Here we present the identification of venom components obtained by sequencing clones isolated from a cDNA library prepared from the venom glands of the Chilean common tarantula, *Grammostola rosea*. The cDNA sequences of about 1500 clones out of 4000 clones were analyzed after selection using several criteria. Forty-eight novel toxin-like peptides (GTx1 to GTx7, and GTx-TCTP and GTx-CRISP) were predicted from the nucleotide sequences. Among these peptides, twenty-four toxins are ICK motif peptides, eleven peptides are MIT1-like peptides, and seven are ESTX-like peptides. Peptides similar to JZTX-64, aptotoxin, CRISP, or TCTP are also obtained. GTx3 series possess a cysteine framework that is conserved among vertebrate MIT1, Bv8, prokineticins, and invertebrate astakines. GTx-CRISP is the first CRISP-like protein identified from the arthropod venom. Real-time PCR revealed that the transcripts for TCTP-like peptide are expressed in both the pereopodal muscle and the venom gland. Furthermore, a unique peptide GTx7-1, whose signal and prepro sequences are essentially identical to those of HaTx1, was obtained.

## 1. Introduction

Venoms are complex mixtures of many different components proven to be useful tools for biochemical, physiological, and pharmacological studies of ion channels and receptors. Toxins that recognize ion channel subgroups are versatile tools for channel studies and thus contribute to drug discovery [1, 2]. For example, a 25-amino-acid peptide isolated from the marine fish-hunting cone snail *Conus magus*, *ω*-conotoxin-MVIIA, blocks N-type voltage-dependent calcium channels. In 2004, ziconotide, the synthetic version of *ω*-conotoxin-MVIIA, was approved in the United States for the treatment of chronic severe pain refractory to other current pain medications.

About 40000 different kinds of spiders are known at present. Spider venoms contain peptide neurotoxins and are expected to be a rich source of ion channel blockers [3–5]. Tarantulas, comprising more than 860 species, like all other spiders are predators that feed on a variety of vertebrate and invertebrate prey [6]. Tarantulas do not use webs for capture but are well-equipped predators, possessing a variety of venoms that target receptors in the nervous system, probably with adaptation to a certain type of prey [7, 8]. Tarantula venom has been suggested to contain 1000 or more peptide toxins [8]. Despite their diverse activities, these toxins display only a few widely conserved structural motifs that share remarkable similarities in their primary sequences and tertiary structures [9–11]. In a similar fashion to the evolution of snake toxins, several molecular scaffolds have been used during the evolution of toxin “cocktails” in spider venoms. The selected genes are duplicated several times, and, while the core of each protein scaffold is conserved, the loops and surfaces are altered through mutations [12]. We recently found T-type voltage-dependent calcium channel blocker from venom of Chilean common tarantula, *Grammostola rosea* [13].

Expressed sequence tags (ESTs) are short single-pass sequence reads generated from either 5′ or 3′ end of cDNAs. They provide a quick and inexpensive route for discovering new genes and obtaining data on gene expression. The ESTs approach has been used in several reports, because it is a rapid and reliable method for gene discovery in general, mainly in this case, related to secretory glands from venomous animals [14–16].

In this paper, we focused on the tarantula toxins, and by applying improved molecular biological techniques, we revealed novel peptide sequences after ESTs techniques applied to a cDNA library prepared from the Chilean common tarantula *Grammostola rosea* venom glands.

## 2. Materials and Methods

### 2.1. Animals and Venom Glands

*Grammostola rosea* tarantulas were obtained from a local pet supplier. The venom glands were dissected from the chelicera and the pereopodal muscles were from the prosoma using sharp forceps, frozen immediately with liquid nitrogen, and then stored at −80°C until use. 

### 2.2. cDNA Library Construction

Preparation of the venom gland cDNA library was reported previously [13]. Briefly, the venom glands were dissected from 30 spiders, and total RNA was extracted using TRIZOL reagent (Invitrogen, Carlsbad, CA). Poly(A)^+^ RNA was prepared using Oligotex-dT30 Super (Takara Bio, Otsu, Japan). The first-strand cDNAs were synthesized from 2.5 *μ*g of poly(A)^+^ RNA using the primer, VNXho(dT)_30_, which installs oligo dT and *Xho*I sequences,  by ReverTra Ace (Toyobo, Osaka, Japan) and Superscript II (Invitrogen). The second strands were synthesized with DNA polymerase I (Takara Bio), RNase H (Takara Bio), and *Escherichia coli* DNA ligase (Takara Bio). *Eco* RI adaptors (Clontech, Palo Alto, CA) were ligated to the cDNAs after both ends of the double-stranded cDNAs were filled in with a DNA blunting kit (Takara Bio). The cDNAs were then digested with *Xho*I and fractionated by 1.2% agarose gel electrophoresis. DNA fragments with lengths of 0.8–2.0 kbp were eluted from dissected gel. The resulting DNA fragments were ligated into *Eco*RI and *Xho*I restriction sites of pSD64TR_ER_ [17]. *E. coli* XL1-Blue MRA (Agilent Technologies, Santa Clara, CA) was transformed with the plasmid. An aliquot of the cDNA library in *E. coli* was spread onto LB agar plates containing 50 *μ*g/mL ampicillin, and the plasmid DNA was prepared for the PCR template.

### 2.3. Fingerprinting of Clones

An aliquot of cDNA library was spread onto LB agar plates with ampicillin (50 *μ* g/mL) and incubated at 37°C overnight. Formed colonies were picked up by automated colony picker (Microtec Nichion, Japan) and inoculated into1 mL of 2 × LB medium supplemented with ampicillin (50 *μ*g/mL) in 96 deep-well plates and incubated at 37°C overnight with vigorous shaking. Plasmids were purified with an automated machine, BIOMEK2000, (Beckman Coulter, USA) using MultiScreen-FB and -NA (Millipore, USA) and eluted by 50 *μ*L TE solution, then stored −20°C until use. PCR was performed with an SP6 primer and a pSD64-specific reverse primer, SDA (5′-TTATGTAGCTTAGAGACT-3′), to amplify the inserts of the cDNA library. Each PCR reaction mixture consists of 10 pmol of the forward and reverse primers, 0.25 U EX Taq polymerase (Takara Bio), 200 mM each of dATP, dCTP, dGTP and dTTP, 2 mM MgCl_2_, PCR buffer, and 1 *μ*L DNA template. The reaction was performed in a thermal cycler PTC-200 (MJ research, USA) for 30 cycles, each consisting of denaturation at 94°C for 30 s, annealing at 42°C for 45 s, and polymerization at 72°C for 1 min, after the initial cycle of 94°C for 5 min. At the end of all the cycles, samples were maintained at 72°C for 9 min and then kept at 4°C.

PCR reaction products were digested by *Dde*I restriction enzyme at 37°C and analyzed by electrophoresis in 3% agarose/TBE gel, then visualized with ethidium bromide, and digitized using gel documentation system, Gel Doc 1000 (Bio-Rad, USA). The band patterns of the digested PCR products were clustered by the similarity using Molecular Analyst Fingerprinting plus software (Bio-Rad).

### 2.4. Sequencing and Data Analysis

We manually selected clones to be sequenced based on the fingerprinting categorization described previously. Single run DNA sequencings were performed using an SP6 primer by a sequencer Model ABI Prism 377 (Applied Biosystems, CA, USA) or performed by Shimadzu. The obtained DNA sequences were translated into amino acid sequences with all three frames using the Vector NTI program (Invitrogen, USA). After translation into three amino acid sequences, both protein and cDNA sequences were stored into in-house database software, KIROKU (World Fusion, Tokyo, Japan). Homology search of translated protein sequences was performed at in-house sequence database using the BLAST program. The prediction of signal sequence was performed by SignalP 3.0 program (http://www.cbs.dtu.dk/services/SignalP/). Amino acid alignment and phylogenetic tree construction were performed using the MegAlign program by Clustal W and neighbor-joining method (DNASTAR, Madison, USA).

### 2.5. PCR Cloning Based on the Signal Sequence of Toxins

We synthesized oligonucleotide primers based on the conserved initiation codon (ATG) and its juxtaposed sequences including 5′-noncoding region and the signal sequences of the GTxs (Table 1). Using these primers and an SDA primer, PCR amplifications were carried out with the venom glands cDNA library as a template. The reaction conditions were essentially the same as described in Section 2.3. Amplified fragments were cloned into pCR 2.1-TOPO (Invitrogen). The full-length nucleotide sequences of the clones were determined by Hitachi Soft Co Ltd.

### 2.6. Quantification of Tissue Expression of GTx-TCTP  and  GTx-CRISP cDNAs

#### 2.6.1. Cloning and Sequence Analysis of GTx-TCTP and GTx-CRISP cDNAs

Single run sequencings of the venom gland cDNA library revealed the first half of GTx-TCTP and GTx-CRISP cDNA including 5′-UTR region with start codon. To obtain the latter halves of GTx-TCTP and GTx-CRISP cDNA including 3′-UTR region with the stop codon from the cDNA library, PCRs were performed using the gene-specific primers, 5′-TCAAGGATATGATTACTGGT-3′ for GTx-TCTP and 5′-AGGTGGGCTGAATCCTGT-3′ for GTx-CRISP, and an SDA primers (see Section 2.3) as forward and reverse primers, respectively. The PCR was carried out in a PTC-200 DNA thermal cycler (MJ Research, South San Francisco, CA) using 30 cycles as follows: denaturation at 94°C for 30 s, annealing at 54°C for 30 s, and extension at 72°C for 1 min using LA Taq polymerase (Takara Bio). The amplified fragments were gel purified using QIAquick Gel Extraction Kit (QIAGEN, Valencia, CA), subcloned into pCR 2.1-TOPO vector (Invitrogen), and sequenced using BigDye Terminator Cycle Sequencing Ready Reaction Kit (version 3.1) and an ABI PRIZM 310 DNA sequencer (Applied Biosystems, Foster City, CA).

#### 2.6.2. Real Time PCR

 The gene expressions of GTx-TCTP and GTx-CRISP in the venom gland and pereopodal muscle were quantified by real-time PCR. Tarantula G3PDH (GTx-G3PDH) cDNA was cloned and used as an expression control. Primers for the real-time PCR were designed using Roche ProbeFinder version 2.45 (http://qpcr.probefinder.com/roche3.html). Primers for GTx-G3PDH were 5′-CATGCTTGGCTAAGGGAGTAA-3′ and 5′-TGTATTTGACATCAATAAATGGATCA-3′; primers for GTx-TCTP, 5′-CTCGGAGAATGGGAGACATT-3′ and 5′-CATCTGCCTCCTCCTGAGAC-3′; and primers for GTx-CRISP, 5′-GCACAATTTCTTCAGGTCACG-3′ and 5′-CAGCTCATTGCCAGCATATC-3′. The venom glands and the pereopodal muscles were stored at −80°C and thawed in TRIZOL reagent (Invitrogen), homogenized, and directly subjected to total RNA extraction according to manufacturer's instructions. Total RNA was reverse transcribed with PrimeScript RT reagent Kit (Takara Bio). Real-time PCR mixtures were prepared with SYBR Premix Ex Taq II (Takara Bio) according to manufacturer's instructions. The reaction and monitoring were performed with Thermal Cycler Dice Real-Time System, PT800 (Takara Bio) for 40 cycles of 2 step shuttle PCR (95°C for 5 s, 60°C for 30 s).

## 3. Results and Discussion

We constructed a tarantula venom gland cDNA library from 2.5 *μ*g of poly (A)^+^ RNA. The independency of the library is about 4.4 × 10^5^. We chose about 1500 clones out of 4000 clones based on the restriction-enzyme digestion patterns determined by fingerprinting software and sequenced by single run sequencing from the upstream region of the protein-coding sequence. After eliminating vector and low-quality sequences, 869 high-quality ESTs were obtained. We found that 284 clones (=32.7%) encode toxin-like sequences among them. This ratio is comparable to the results from the EST analysis of the venom glands of Theraphosidae family tarantulas *Chilobrachys jingzhao* and *Citharischius crawshayi*, in which 30.6% and 32.5% of analyzed clones encode toxin-like sequences, respectively [16, 18, 19]. In the present study, eight types of toxin-like scaffold were found mainly based on a cysteine framework. It is noteworthy that 15 and 5 peptide scaffolds were reported from the EST studies of the tarantulas *Chilobrachys jingzhao* and *Citharischius crawshayi*, respectively, [16, 18, 19]. Thirty-four cDNAs were additionally revealed by PCR cloning using primers designed from the conserved initiation codon (ATG) and its juxtaposed sequences including 5′-noncoding region and the signal sequences. 

We focused on unique 48 peptides belonging to eight types of toxin-like scaffold. The resulted sequence analysis and the database search are described here in after.

### 3.1. GTx1 Family, Long Loop ICK Motif Toxins; and GTx2 Family, Short Loop ICK Motif Toxins

**Figure 1:**
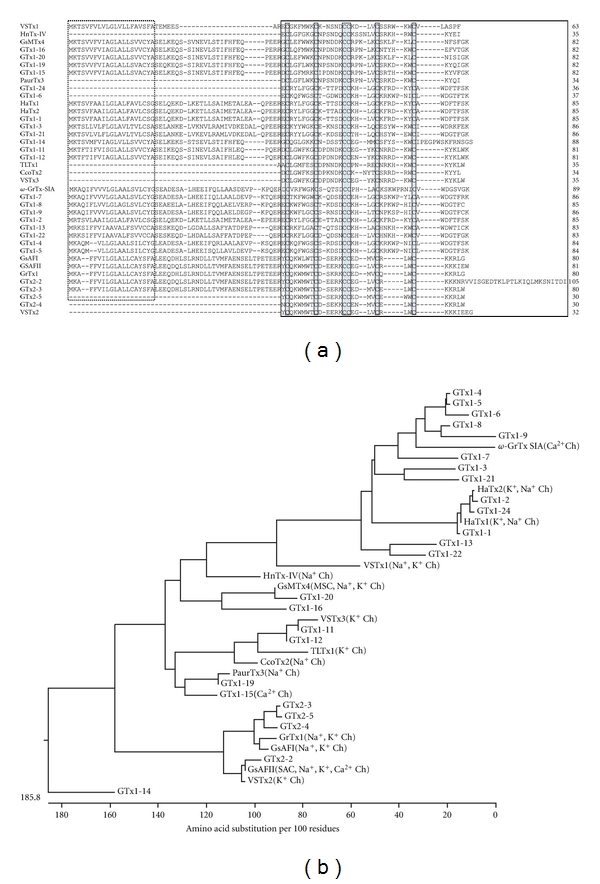
Homology analysis of ICK motif toxins. (a) Homology alignment of ICKs. The putative signal sequences deduced by SignalP 3.0 server (http://www.cbs.dtu.dk/services/SignalP/) are indicated by dotted box. Signal sequences and prepro-sequences of GTx1-6, GTx1-24, GTx2-4, GTx2-5, HnTx-IV, PaurTx3, TLTx1, CcoTx2, and VSTx1, 2, 3 are not determined. Mature toxin regions are indicated by closed box. Conserved cysteine residues are indicated by closed boxes filled with gray color. (b) Phylogenetic tree of ICK toxins. Amino acid alignment and phylogenetic tree construction were performed using the MegAlign program by Clustal W and neighbor-joining method (DNASTAR, Madison, USA) based on the alignment (a) of mature peptides. A scale below the tree indicates the number of amino acid substitutions per 100 residues for protein sequences. Known molecular targets are indicated following to the peptide names. Accession numbers: GTx1 and GTx2 families, AB200996-AB201024 and AB671302-AB671308; HaTx1, AB200991; HaTx2, AB200992; VSTx1, AB200994; GsAFI, AB200995; GsAFII, AB612242; GrTx1, AB671300 and AB671301; *ω*-GrTx SIA, AB612243; GsMTx4, AB201020; PaurTx3, P84510; CcoTx2, P84508; TLTx1, P83745; VSTx2, P0C2P4; VSTx3, P0C2P5.

Twenty long loop ICK motif toxins and four short loop ICK motif toxins were obtained (Figure 1(a)). In the mature sequences, the N-terminal and the C-terminal sequence stretches separated by cysteine residues are termed “loops” and numbered 1–6 from N- to C-terminus. The toxins with more than 6 amino acids and the toxins with only three amino acids in the loop 5 are designated as “long-loop” and “short-loop” ICK toxins group, respectively. Among these toxins, we previously showed that GTx1-15 preferentially block the currents of the T-type voltage-dependent calcium channel Ca_v_3.1 [13]. Among the GTx1 and GTx2 families, we identified additional peptide cDNAs; those translated sequences are the same as the previously reported ones, HaTx1 and 2, VSTx1 and 2, GsMTx4, GsAFI and II, GrTx1, and *ω*-GrTx SIA. HaTx1 and 2 are well-known toxins that inhibit K_v_2.1 and K_v_4.2 voltage-gated potassium channels [20]. VSTx1 is a voltage sensor toxin from the spider *Grammostola rosea* that inhibits K_v_AP, an archeabacterial voltage-activated potassium channel whose X-ray structure has been reported [21]. GsMTx4 is known as a toxin for stretch-activated mechanosensitive channels [22]. GsAFI and II have been first reported to be an analgesic and an antiarrhythmic peptides from the venom of spider *Grammostola rosea*, respectively [23, 24]. GsAFI, GsAFII, and GrTx1 have been shown to have similar blockade spectra against ion channels such as Na_v_1.1, Na_v_1.2, Na_v_1.3, Na_v_1.4, Na_v_1.6, Na_v_1.7, and K_v_11.1 [25, 26]. *ω*-GrTx SIA is reported to inhibit Ca_v_2.1 and Ca_v_2.2 voltage-dependent calcium channels by modifying their voltage-dependent gating [27, 28].

As mentioned previously, HaTx1 is known as a potassium channel gating modifier but can inhibit sodium channels at concentrations similar to those that modify the gating of potassium channels [29]. Recently, similar target promiscuity and heterogeneous effects of tarantula venom voltage-sensor toxins are discussed including GsAFI and II, GrTx1 described previously [25, 30]. We also suggest similar target promiscuity; that is, a toxin family including GsAFII, GsMTx2, PaTx2, and ProTx-II could affect several types of ion channels such as stretch-activated channels and the voltage-dependent sodium, potassium, and calcium channels [13].  Figure 1(b) shows phylogenetic tree of GTx1, GTx2, and several ICK toxins. The correlations between the peptide groups and their target molecules are not clear. Vega discussed the pharmacological diversification of ICK motif toxin by phylogenetic analysis of 171 homologous ICK toxins using Bayesian inference [31]. Although the relationship between clusters is not satisfactorily solved, several trustable monophyletic groups appear from the analysis. The main conclusion from the tree is a plausible linage-specific process of paralogous diversification from several independent recruiting events. Further investigation is needed to elucidate the relationships between evolutional processes and the pharmacological diversification and target promiscuity of ICK toxins.

### 3.2. GTx3 Series: Similar to Mamba Intestinal Toxin 1 (MIT1), Bv8/Prokineticins, and Invertebrate Astakines

**Figure 2:**
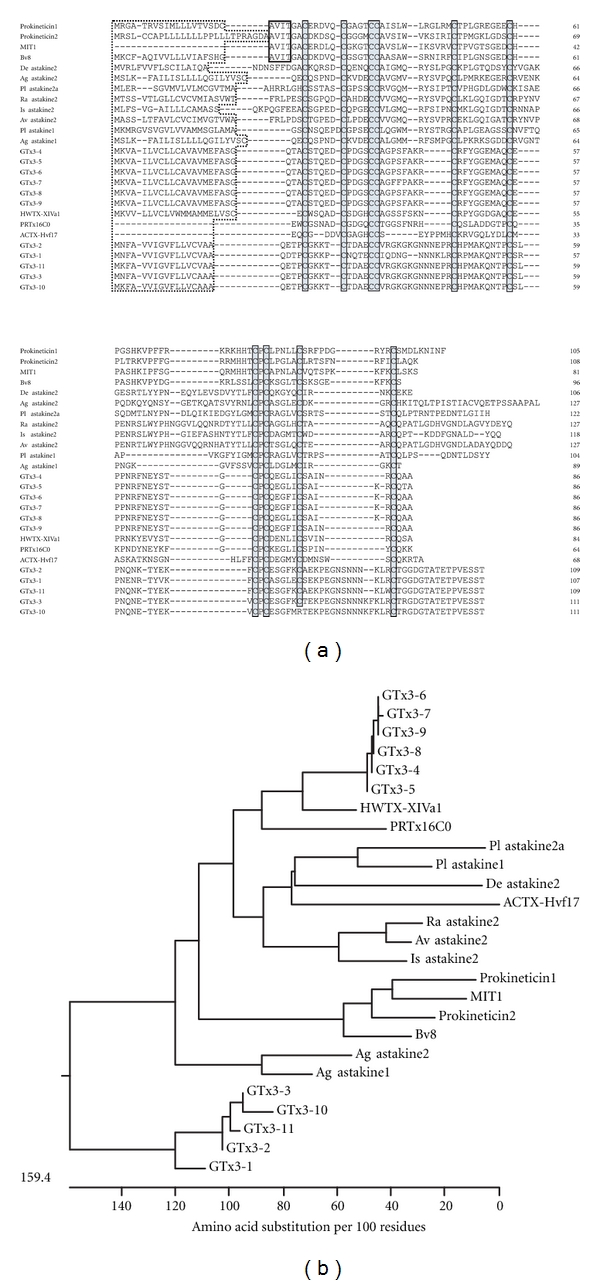
Homology analysis of GTx3 series toxins. (a) Sequence alignment of GTx3 series and their related peptides. The putative signal sequence deduced by SignalP 3.0 server (http://www.cbs.dtu.dk/services/SignalP/) is indicated by dotted box. Signal sequence of MIT1 is not determined. Conserved cysteine residues are indicated by closed boxes filled with gray color. AVIT N-terminals of Prokineticin1, Prokineticin2, MIT1, and Bv8 are indicated closed boxes. (b) Phylogenetic tree of GTx3 series and related peptides. Amino acid alignment and phylogenetic tree construction were performed using the MegAlign program by Clustal W and neighbor-joining method (DNASTAR) based on the alignment (a) of mature peptides. A scale below the tree indicates the number of amino acid substitutions per 100 residues for protein sequences. Accession numbers: GTx3 series, AB201025-AB201034 and AB671309-AB671311; Bv8, AF168790; Prokineticin 1, AF333024; Prokineticin 2, AAH96695; MIT1, P25687; HWTX-XIVa1, ABY77690; ACTX-Hvf17, P81803; *Pacifastacus leniusculus* (Pl) astakine1, AY787656; Pl astakine2a, EF568370; *Acanthoscurria gomesiana* (Ag) astakine1, DR447331; Ag astakine2, DR445103; *Rhipicephalus appendiculatus* (Ra) astakine2, CD794853; *Ixodes scapularis* (Is) astakine2, EW845057; *Amblyomma variegatum* (Av) astakine2, BM292046; *Dysdera erythrina* (De) astakine2, CV178181.

Eleven toxins similar to MIT1, Bv8/prokineticins, and invertebrate astakines were identified (Figure 2). Bv8, prokineticins, and MIT1 consist in a group known as AVIT family due to their N-terminal residues A-V-I-T [32]. MIT1 shows contractile effects on longitudinal ileal muscle and distal colon [33]. The solution structure of MIT1 was determined at a resolution of 0.5 Å and revealed a new type of folding for venom toxins similar to that of colipase, a protein involved in fatty acid digestion [34]. Bv8 is bioactive peptide found from frog skin to induce hyperalgesic effects [35] and belongs to a family of secretory proteins (Bv8-prokineticin family) whose orthologues have been conserved throughout evolution from invertebrates to human. The prokineticins (PK1 and PK2, also known as endocrine gland vascular endothelial growth factor (EG-VEGF) and Bv8, resp.) are involved in signaling through two highly homologous G-protein-coupled receptors, PKR1 and PKR2 [36]. Bv8/PK2 is upregulated in inflammatory granulocytes and modulates inflammatory pain [37]. Blockade of PKRs might represent a therapeutic strategy in acute and inflammatory pains [38].

Vertebrate PKs are released from damaged tissues and act as regulators of inflammatory responses, including recruitment of new blood cells [39]. Invertebrate astakine, a homologue to vertebrate PKs, was first identified in *Pacifastacus leniusculus* and was found to be necessary for new hemocyte synthesis and release [40]. Although astakines lack the N-terminal AVIT motif, they are designated as prokineticin domain-containing proteins based on their hematopoietic function. No astakine or prokineticin homologue is present in the genome of *Drosophila *or other dipterans, so far.  Figure 2(a) shows that the cysteine frameworks of GTx3 series peptides, vertebrate PKs, invertebrate astakines, and several peptide toxins are well conserved. ACTX-Hvf17 from Australian funnel-web spiders lacks the N-terminal AVIT motif and did not affect smooth muscle contractility or block PK1-induced contractions in guinea pig ileum [5]. PRTx16C0 from Brazilian Amazonian armed spider (accession no. P83893) is nontoxic to mice and insects. The effect of HWTX-XIVa1 from Chinese bird spider is unknown [41]. Phyogenetic tree shows that GTx3-4 to GTx3-9, invertebrate astakines, vertebrate prokineticin-related proteins, and spider toxins form large family, while GTx3-1 to GTx3-3, GTx3-10 and GTx3-11 form a distantly related group (Figure 2(b)). For the spider proteins containing prokineticin domain (ACTX-Hvf17, PRTx16C0, HWTX-XIVa1, and GTx3 series), further investigation is needed to reveal their biological functions, especially their effects on the hematopoietic system. 

### 3.3. GTx4, 5, 6 Series: Similar to Other Toxins

**Figure 3:**
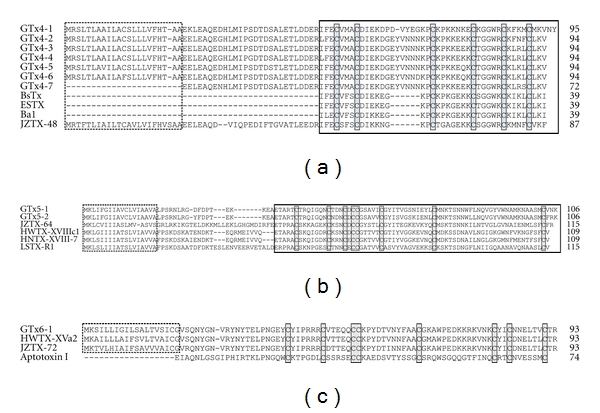
Sequence alignments of GTx4 (a), GTx5 (b), and GTx6 (c) series. The putative signal sequence deduced by SignalP 3.0 server (http://www.cbs.dtu.dk/services/SignalP/) is indicated by dotted box. Signal sequences of GTx4-7 and signal sequences and prepro-sequences of BsTx, ESTX, and Ba1 are not determined. Mature toxin regions are indicated by closed box in (a) and (b). Conserved cysteine residues are indicated by closed boxes filled with gray color. Accession numbers: GTx4 series, AB201035-AB201036 and AB671312-AB671317; BsTx, P49265; ESTX, P61509; Ba1, P85497; JZTX-48, EU233840; GTx5-1 and GTx5-2, AB201037 and AB683255; JZTX-64, EU233914; HWTX-XVIIIc1, EU195231; HNTX-XVIII-7, GU293118; LSTX-R1, EU926143; GTx6-1, AB201038; HWTX-XVa2, EU195236; JZTX-72, EU233926; Aptotoxin I, P49267.

GTx4 series are similar to ESTX [42], BsTx [43], JZTX-47, 48 [16], and Ba1, 2 [44] (Figure 3(a)). They are characterized as conserved six cysteine residues. A clear difference between the sequences of GTx4 series and that of the reported ones is the length of the loop 3. ESTX is purified from tarantula *Eurypelma californicum* venom and BsTx is from Mexican red nee tarantula *Brachypelma smithii* venom. The effects of ESTX and BsTx are not clear. Ba1 and Ba2 are insecticidal peptides purified from theraphosid spider *Brachypelma albiceps* venom and an NMR-based 3D model of Ba2 is proposed [44].

GTx5-1 and GTx5-2 are similar to JZTX-64 from *Chilobrachys jingzhao* [16], HWTX-XVIIIc1 from *Ornithoctonus huwena* [41], HNTX-XVIII-7 from *Ornithoctonus hainana* [45], and LSTX-R1 from *Lycosa singoriensis* [46] (Figure 3(b)). These toxins are identified by large-scale venomic strategy and the target molecules are unknown.

GTx6-1 is very similar to HWTX-XVa2 from *Haplopelma schmidti* [41] and JZTX-72 from *Chilobrachys guangxiensis* [16], and similar to aptotoxin I [47], as well (Figure 3(c)).

As mentioned previously, insecticidal effects were reported for Ba1, Ba2, and aptotoxin; however, target molecules of GTx4, 5, 6, and their homologues are not yet known.

### 3.4. GTx-TCTP and GTx-CRISP

**Figure 4:**
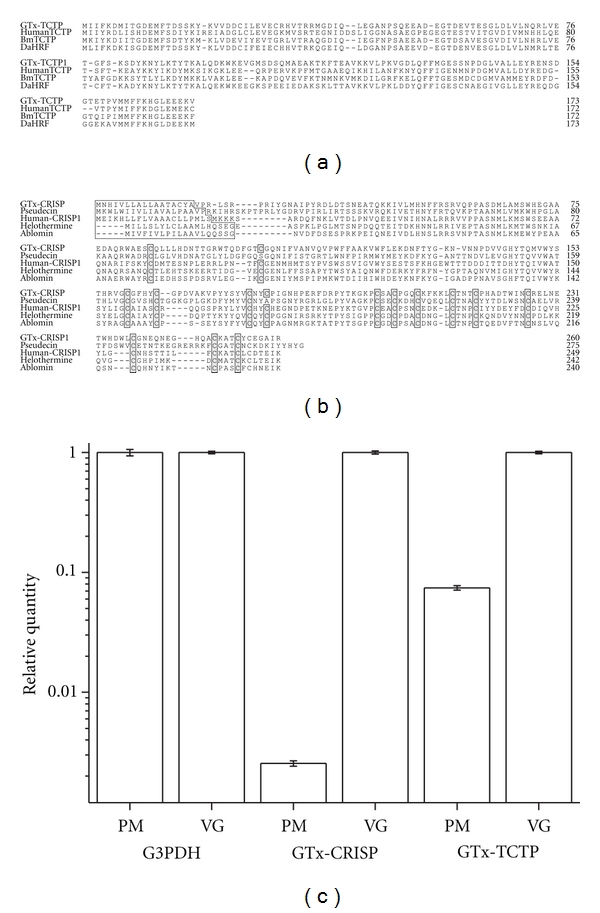
Sequence alignment of GTx-TCTP (a) and GTx-CRISP (b) families. The putative signal sequence deduced by SignalP 3.0 server (http://www.cbs.dtu.dk/services/SignalP/) is indicated by dotted box. Conserved cysteine residues are indicated by closed boxes filled with gray color. (c) The results of real-time PCR. G3PDH is equally expressed in the pereopodal muscle (PM) and the venom gland (VG). GTx-CRISP transcript is predominantly expressed in the venom gland. The GTx-CRISP transcript in the PM is one-400th of that in the VG. GTx-TCTP transcript is expressed in both the pereopodal muscle and the venom gland. The GTx-TCTP transcript in the PM is one-13th of that in the VG. Results were confirmed in triplicate experiments. Accession numbers: GTx-TCTP, AB201040; HumanTCTP, NM_003295; *Bombyx mori* (Bm) TCTP, NM_001044107; *Dermacentor andersoni *(Da) HRF, DQ009480; GTx-CRISP, AB201041; Pseudecin (*Harpegnathos saltator*), EFN80524; Human-CRISP1, NM_001205220; Helothermine, U13619; Ablomin, AF384218.

We also obtained one translationally controlled tumor protein- (TCTP-) like peptide (Figure 4(a)) and one cysteine-rich secretory protein-(CRISP-) like peptide (Figure 4(b)).

 TCTP was first identified as a growth-related tumor protein whose synthesis is controlled mainly at the translational level [48]. This protein has been recognized as a cell cycle-dependent, tubulin-binding protein having calcium-binding sites [49]. In addition to this growth-related function as a cytosolic protein, TCTP is now known to act as a secretory protein. TCTP has been uniquely characterized as an IgE-dependent histamine-releasing factor [50].

CRISPs are found in a variety of organisms, such as mammals, reptiles, amphibians, and secernentea. The first discovered CRISP (acidic epididymis glycoprotein, also known as protein D/E or CRISP-1) was isolated from mammalian epididymis [51–53]. Two other mammalian CRISPs have been isolated and characterized: CRISP-2 (testis-specific protein 1) [54] and CRISP-3 (specific granule protein of 28 kDa) [55]. Venomic CRISPs were identified mainly from lizard and snake, so far. Helothermine, a CRISP family toxin, is discovered from the lizard of the Central America [56] and blocks voltage-gated calcium and potassium channels and ryanodine receptors [57]. Ablomin is a 25-kDa protein isolated from the venom of the Japanese Mamushi snake (*Agkistrodon blomhoffi*) [58]. Ablomin blocks contraction of rat tail arterial smooth muscle elicited by high K^+^-induced depolarization. In insects, it is revealed that an ant (*Harpegnathos saltator*) genome contains a CRISP family protein, Pseudecin [59]. GTx-CRISP is the first CRISP protein identified from the arthropod venom.

To compare the expression levels of the transcripts for GTx-CRISP and GTx-TCTP between the venom gland and the pereopodal muscle, we conducted real-time PCR. The results indicate that transcript of GTx-TCTP was expressed in both the tissues, while that of GTx-CRISP was predominantly expressed in the venom gland (Figure 4(c)). It is tempting to assume that GTx-TCTP acts as both growth-related cytosolic protein and secretory proteins, an IgE-dependent histamine-releasing factor. Further investigation is needed to elucidate the bifunctional feature of GTx-TCTP.

### 3.5. Other Toxins

**Figure 5:**
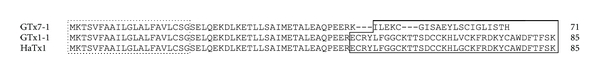
Sequence alignment of GTx7-1. The putative signal sequence deduced by SignalP 3.0 server (http://www.cbs.dtu.dk/services/SignalP/) is indicated by dotted box. Mature toxin regions are indicated by closed box. Note that the signal sequences and prepro-sequences are almost same but mature GTx7-1 differs from GTx1-1 and HaTx1. Accession number: GTx7-1, AB201039.

The predicted mature portion of the peptide GTx7-1, 21 amino acid residues with two cysteine residues, has a unique sequence (Figure 5). It has no amino acid sequence homology with any other peptide registered in the public database up to now. On the other hand, the preprotoxin sequence of GTx7-1 is very similar to GTx1 family. GTx7-1 slightly and transiently inhibited the contraction of guinea pig right atrial preparation at high concentration (13.2 *μ*M) (Japan patent publication number: 2008-271800).

### 3.6. Biochemical and Biomedical Applications of Peptide Toxins

 Natural peptide toxins contribute to biochemical, physiological, and pharmacological studies especially on cellular/neuronal signal transduction. Furthermore, some of the peptides and its derivatives have been developed as potential therapeutic agents. Their utility is based on an unprecedented selectivity in targeting specific molecular forms, such as subgroups of ion channels and subtypes of receptors, and even specific substates of channel functions.

 There are principally two approaches to access to the peptide with some aimed function/property. One is to screen such a peptide from the venom/secreta or tissue extracts, and the other is to screen from cDNA libraries followed by expression and functional assays. Recently, we have developed a new approach by utilizing natural toxin scaffold combined with *in vitro* selection technology. We first constructed a random peptide library based on a three-finger (3F) neurotoxin scaffold. From the 3F peptide library, *in vitro* selections targeting to interleukin-6 receptor were performed, and finally peptide ligands with the antagonist-like and the agonist-like property were generated [60]. Variety of toxin scaffolds are available up to now, and still unknown scaffolds might be revealed by genomic approach for the venom/secretion glands. The new *in vitro* evolution approach will be further applied to different toxin scaffolds including ICK motifs and also will be directed to different targets, such as biomarkers for diagnosis or drug development, or target cells for imaging and drug delivery, and so forth.

## 4. Conclusions

 We have challenged to reveal a peptide repertoire contained in the venom gland of tarantula *Grammostola rosea*. In the previous report, we identified several novel peptides from the spider venom by both proteomic and genomic approaches and reported their modulation activities toward calcium channels [13]. Here in this study, we further presented 48 novel peptides grouped as GTx1 to GTx7, and TCTP- and CRISP-like peptides, and compared the sequences with the homologues. GTx1 and 2 series are mostly homologous to ion channel blockers. GTx3 series are related to the peptides that modulate cell growth and/or cell signaling via GPCRs such as vertebrate prokineticins and invertebrate astakines. GTx-CRISP is the first identified arthropod venom CRISP. GTx-TCTP is expressed in both the venom gland and the pereopodal muscle and assumed to act as both TCTP and HRF. Biochemical and physiological characterizations of these peptides are under investigation. Furthermore, we are now applying the next generation sequencing to totally reveal the transcripts of the tarantula venom gland. 

## Figures and Tables

**Table 1:** Primers for PCR cloning.

Primers	Nucleotide sequence	Referred sequences
PC1	5′-TAARCGACAATGAAGAC-3′	GTx1-11, 12, 14, 15, 16
PC2	5′-TTCGATAACATGAAGAC-3′	GTx1-1, 2; GTx7-1
PC3	5′-AAAGCATGAAAACCTC-3′	GTx1-3
PC4	5′-ACTCTAAAAATGAAGGC-3′	GTx1-4, 5, 7, 8, 9
PC5	5′-TCAGCAGAAATGAAGGC-3′	GTx2-2, 3
PC6	5′-TCCATCATGAAGITNGC-3′	GTx3-4, 5, 6, 7, 8
PC7	5′-ATAACGATGAAGITINT-3′	GTx5-1
PC8	5′-GCAGCCATGAAAICINT-3′	GTx6-1
PC9	5′-GTTAAGATGAAITWYNC-3′	GTx3-1, 2, 3
PC10	5′-GCAACGATGAGRTCINT-3′	GTx4-1, 2
PC11	5′-GGAAACATGAGRAAINC-3′	GTx1-13

Oligonucleotide primers are synthesized based on the conserved signal sequences and the sequcences of 5–9 nucleotides upstream of initiation codon of the GTxs indicated in the right column. Underline indicates initiation codon, ATG. R: A/G; W: T/A; Y: T/C; N: A/T/C/G; I: inosine.
